# Predictive Value of the Systemic Immune‐Inflammation Index for the Clinical Efficacy of Acupuncture and Exercise Rehabilitation in Knee Osteoarthritis: A Retrospective Study

**DOI:** 10.1002/iid3.70430

**Published:** 2026-05-06

**Authors:** Shifei Sun, Zhiyong Lu, Qing Yang, Wenchang Xu, Fengjun Zhang, Gongchang Yu, Bin Shi

**Affiliations:** ^1^ School of Acupuncture and Tuina Shandong University of Traditional Chinese Medicine Jinan China; ^2^ Department of Traditional Chinese Orthopedics and Traumatology, Shandong Key Laboratory of Biomechanics Neck‐Shoulder and Lumbocrural Pain Hospital of Shandong First Medical University, Shandong First Medical University & Shandong Academy of Medical Sciences Jinan China; ^3^ Department of Hematology The 960th Hospital of the Joint Logistics Support Force of the Chinese People's Liberation Army Jinan China; ^4^ Department of Traditional Chinese Orthopedics and Traumatology First Clinical Medical College of Shandong University of Traditional Chinese Medicine Jinan China; ^5^ Department of Pulmonary Diseases ShuGuang Hospital, Shanghai University of Traditional Chinese Medicine Shanghai China; ^6^ Department of Science and Education, Shandong Key Laboratory of Biomechanics Neck‐Shoulder and Lumbocrural Pain Hospital of Shandong First Medical University, Shandong First Medical University & Shandong Academy of Medical Sciences Jinan China

**Keywords:** acupuncture, exercise rehabilitation, knee osteoarthritis, predictive biomarker, systemic immune‐inflammation index, treatment response

## Abstract

**Objective:**

To evaluate the predictive value of the systemic immune‐inflammation index (SII) for clinical efficacy of acupuncture combined with exercise rehabilitation in patients with knee osteoarthritis (KOA).

**Methods:**

In this retrospective observational study, 151 patients with radiographically confirmed Kellgren–Lawrence Grade II or III KOA who completed an 8‐week standardized protocol of acupuncture and exercise rehabilitation were enrolled. Clinical efficacy was defined as the attainment of minimal clinically important improvement (MCII) in both the 11‐point Numeric Rating Scale (NRS) for pain (≥ 2‐point reduction) and the 68‐point WOMAC function subscale (≥ 6‐point improvement, Likert version 3.1). Baseline demographic, clinical, and hematological data were collected, and SII was subsequently calculated from baseline hematological parameters. Uni‐ and multivariate logistic regression analyses were used to identify independent predictors of treatment response. Receiver operating characteristic (ROC) curves were constructed to assess the discriminative ability of SII, body mass index (BMI), and lymphocyte count, individually and in combination.

**Results:**

Of the 151 patients, 73 were classified as responders and 78 as nonresponders. Baseline BMI and SII were significantly lower, while lymphocyte count was higher in the effective group (all *p* < 0.001). Multivariate logistic regression identified BMI (OR = 0.596, 95% CI: 0.472–0.753), lymphocyte count (OR = 34.597, 95% CI: 3.234–370.131), and SII (OR = 0.912, 95% CI: 0.873–0.953) as independent predictors of treatment response. ROC analysis showed that SII demonstrated only moderate predictive power (AUC = 0.749, 95% CI: 0.670–0.828), with improved but still moderate accuracy when combined with BMI and lymphocyte count (AUC = 0.858, 95% CI: 0.799–0.917).

**Conclusion:**

Although SII is an independent and accessible biomarker for predicting clinical efficacy, its discriminative ability is moderate. A combined model incorporating SII, BMI, and lymphocyte count may provide better, though not strong, predictive performance and could support clinical decision‐making and individualized treatment planning. However, the retrospective single‐center design may introduce selection bias and limit generalizability. Residual confounding from unmeasured variables cannot be excluded, and the absence of longitudinal SII measurements limits evaluation of temporal changes. Future multicenter prospective studies are warranted to validate these findings.

## Summary

- SII represents a relatively practical and readily obtainable marker for estimating clinical efficacy in KOA patients undergoing acupuncture and exercise rehabilitation.
- Together with BMI and lymphocyte count, SII offers a promising biomarker for stratifying patients, optimizing therapeutic strategies, and improving clinical outcomes.
- Systemic inflammatory and metabolic status in determining treatment responsiveness and provide a foundation for integrating biological markers into individualized care pathways for KOA.

**Abbreviations**
ACR: American College of Rheumatology
BMI: body mass index
CI: confidence intervals
KOA: knee osteoarthritis
MCII: minimal clinically important improvement
NRS: Numeric Rating Scale
OARSI: Osteoarthritis Research Society International
OR: odds ratios
ROC: receiver operating characteristic
SII: systemic immune‐inflammation index

## 1 Introduction

Knee osteoarthritis (KOA) is a progressive degenerative joint disorder and one of the most prevalent causes of chronic pain and functional disability among middle‐aged and older adults [1, 2]. Globally, KOA affects approximately 250 million people and is a major contributor to reduced mobility, impaired quality of life, and increased socioeconomic burden [3]. The pathological hallmarks of KOA include articular cartilage degradation, subchondral bone sclerosis, osteophyte formation, and varying degrees of synovial inflammation [4, 5]. While the etiology of KOA is multifactorial, low‐grade systemic inflammation has been increasingly recognized as a key contributor to both disease initiation and progression.

Current therapeutic strategies for KOA primarily focus on symptom management, as no disease‐modifying treatments are yet available. Nonpharmacological interventions, including physical therapy and lifestyle modification, remain the cornerstone of early and mid‐stage KOA management. Acupuncture, rooted in Traditional Chinese Medicine (TCM), is guided by pattern (syndrome) differentiation, with KOA most commonly classified under “Bi syndrome” (impediment syndrome) caused by wind, cold, dampness, or heat obstructing the meridians and leading to qi and blood stagnation in the knee. Treatment principles focus on dispelling pathogenic factors, promoting qi and blood circulation, and alleviating pain. In the context of KOA, it is believed to exert therapeutic effects through multiple mechanisms, including modulation of inflammatory cytokines (e.g., TNF‐α, IL‐1β, IL‐6), suppression of synovial hyperplasia, enhancement of endogenous opioid release for analgesia, and regulation of central pain processing pathways [6, 7, 8, 9, 10]. Neuroimaging studies have further demonstrated that acupuncture can influence brain regions associated with pain perception and motor control, while experimental data suggest it may improve local microcirculation and promote cartilage‐protective effects [11]. Clinical guidelines, including those from the American College of Rheumatology (ACR) and Osteoarthritis Research Society International (OARSI), endorse acupuncture and exercise therapy as part of a multimodal treatment approach [12, 13]. Concurrently, structured exercise rehabilitation targeting joint stability, muscular strength, and neuromuscular coordination has shown considerable efficacy in improving pain and function [14, 15]. The combination of acupuncture with exercise therapy offers a synergistic modality, yet variability in individual treatment responses limits its widespread optimization. However, most KOA outcome assessments, such as the NRS and WOMAC, are subjective and prone to recall bias, mood effects, and individual differences in pain perception, potentially leading to variability and misclassification of treatment response. They may also fail to detect subclinical inflammatory or structural changes, underscoring the need for objective, quantifiable, and reproducible biomarkers to complement patient‐reported outcomes and improve evaluation accuracy.

Given the heterogeneity in clinical outcomes, there is a growing imperative to identify objective and cost‐efficient biomarkers capable of predicting treatment response. Such predictive tools would allow for personalized treatment planning, early identification of likely responders, and more efficient allocation of healthcare resources. In this context, biomarkers derived from routine blood tests, which are minimally invasive and readily available in clinical practice, are of particular interest. The systemic immune‐inflammation index (SII), calculated as platelet count × neutrophil count/lymphocyte count, integrates information from three major immune cell lines and reflects both inflammatory and immune status [16, 17]. Originally proposed as a prognostic indicator in oncology [18], SII has subsequently demonstrated relevance in cardiovascular disease [19], autoimmune disorders [20], and several chronic inflammatory conditions [21].

In the specific context of KOA, multiple studies have shown that peripheral inflammatory cell ratios, such as neutrophil‐to‐lymphocyte ratio (NLR) and platelet‐to‐lymphocyte ratio (PLR), are associated with radiographic severity, cartilage degeneration, and symptom intensity [22, 23, 24]. SII incorporates both NLR and PLR components, thereby providing a more comprehensive measure of systemic immune‐inflammatory balance. This composite nature may offer superior sensitivity in detecting subtle inflammatory alterations that influence pain perception, synovial activity, and overall treatment responsiveness. Furthermore, acupuncture and exercise rehabilitation have been reported to modulate systemic inflammatory mediators and immune cell profiles in KOA patients [25, 26], suggesting a plausible mechanistic link whereby baseline SII could help identify individuals more likely to benefit from these interventions.

In osteoarthritis, elevated systemic inflammatory markers have been associated with disease severity, joint degradation, and symptom burden [27]; however, to the best of our knowledge, no study has examined the predictive role of SII in acupuncture, either alone or in combination with exercise rehabilitation, making this the first investigation in the acupuncture field and highlighting its novelty in addressing a research gap and providing preliminary evidence for incorporating an immune‐inflammatory biomarker into conservative KOA treatment planning. Accordingly, this study aimed to assess the predictive utility of baseline SII in evaluating the clinical efficacy of acupuncture combined with exercise rehabilitation in patients with KOA. We hypothesized that lower baseline SII would be associated with a greater likelihood of achieving clinically meaningful improvement in pain and function following an 8‐week program of acupuncture combined with exercise rehabilitation.

## 2 Methods

### 2.1 Participants Enrollment

**Figure 1:**
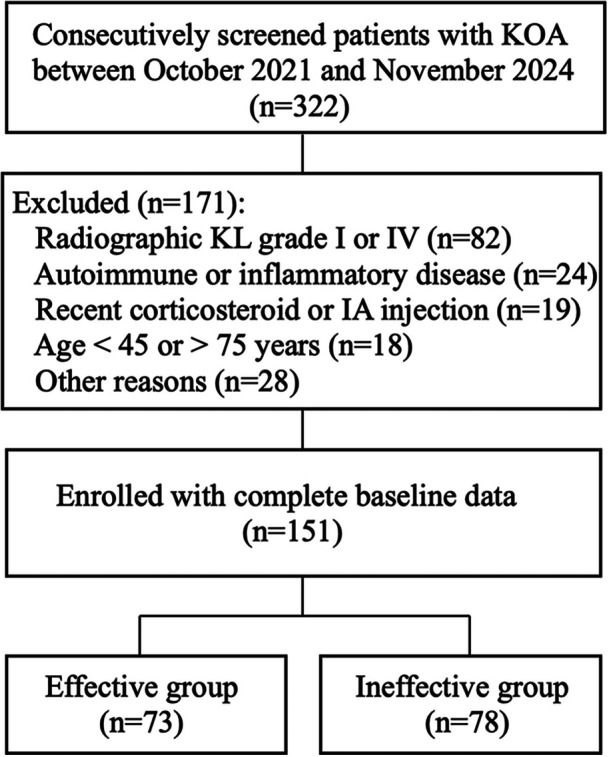
Flowchart illustrating the patient selection process.

This retrospective observational study was conducted at Neck‐Shoulder and Lumbocrural Pain Hospital of Shandong First Medical University, a tertiary care center specializing in musculoskeletal disorders. A total of 322 patients with KOA who visited the hospital between October 2021 and November 2024 were consecutively screened. Of these, 171 were excluded (radiographic KL Grade I or IV, autoimmune or inflammatory disease, recent corticosteroid or intra‐articular injection, age < 45 or > 75 years, or other reasons). The remaining 151 patients, all with complete baseline and outcome data, were enrolled, as detailed in the updated screening flowchart (Figure 1). All patients fulfilled the diagnostic criteria for KOA established by the ACR [28] and had radiographic evidence consistent with Kellgren–Lawrence Grade II or III changes [29], which were selected to represent patients with mild‐to‐moderate structural damage. Grade I cases were excluded to avoid diagnostic uncertainty and the potential inclusion of individuals with minimal or nonspecific radiographic changes, while Grade IV cases were excluded due to advanced joint destruction, which is less likely to benefit from conservative interventions such as acupuncture and exercise rehabilitation. Inclusion criteria: (1) chronic knee pain lasting ≥ 6 months; (2) age between 45 and 75 years; (3) completion of a standardized acupuncture and exercise rehabilitation protocol for at least 8 weeks; and (4) intact baseline clinical and laboratory data. Exclusion criteria included the presence of autoimmune or systemic inflammatory diseases, malignancy, active infection, or recent administration of corticosteroids or immunosuppressive medications or prior intra‐articular injection within 3 months. Eligibility was independently verified by two senior clinicians, and any discrepancies were resolved through discussion to ensure strict adherence to the inclusion and exclusion criteria.

All patients received an integrative treatment regimen comprising traditional manual acupuncture delivered by licensed acupuncturists (three 30‐min sessions per week) combined with personalized exercise rehabilitation targeting joint mobility, muscle strength, and neuromuscular control. Treatment response was evaluated at 8 weeks postintervention. This study was conducted in accordance with the principles of the Declaration of Helsinki and the protocol was obtained by the Ethics Committee of Neck‐Shoulder and Lumbocrural Pain Hospital of Shandong First Medical University. All participants signed written informed consent.

### 2.2 Clinical Response and Study Design

The clinical response was defined based on the attainment of minimal clinically important improvement (MCII) on both the 11‐point Numeric Rating Scale (NRS) [30] for pain and the 68‐point WOMAC function subscale (Likert version 3.1) [31]. Specifically, the MCII was set at a 2‐point reduction on the NRS, extrapolated from a 19.9‐mm change on the 100‐mm Visual Analog Scale, and a 6‐point improvement on the WOMAC function subscale, extrapolated from a 9.1‐point change on the normalized 100‐point scale [32, 33]. Patients who met both criteria were classified as responders (i.e., effective group, *n* = 73), while those who did not meet both thresholds were considered nonresponders (i.e., ineffective group, *n* = 78). To minimize assessment bias, all outcome evaluations were conducted by two independent assessors who were blinded to the patients' group allocation, baseline SII levels, and all other laboratory results. Both assessors underwent prestudy training to standardize scoring procedures, and discrepancies in scoring were resolved by consensus. A priori sample size calculation was not performed because this was a retrospective observational study using all eligible patients admitted during the study period. However, a post hoc power analysis based on the observed effect size for the primary predictor (SII) and the actual sample size (*n* = 151) indicated a statistical power exceeding 0.80 at *α* = 0.05, suggesting that the study was adequately powered to detect meaningful associations.

### 2.3 Treatment

All enrolled patients underwent an 8‐week standardized intervention protocol consisting of acupuncture combined with exercise rehabilitation. Treatment sessions were administered once daily throughout the study period. Acupuncture was performed by licensed practitioners using sterile, single‐use disposable needles. Patients were positioned in the supine posture, and acupuncture was applied at standardized acupoints commonly used in the treatment of KOA, including Liangqiu (ST34), Heding (EX‐LE2), Ashi points, Xiyan (EX‐LE5), Yinlingquan (SP9), Xuehai (SP10), and Yanglingquan (GB34) [34, 35]. The balanced reinforcing and reducing technique (Ping Bu Ping Xie) with manual twirling manipulation was employed [34, 35]. Each treatment session lasted 15–20 min. No electroacupuncture or additional adjunctive therapies were applied. To minimize potential inter‐practitioner variability, all acupuncture procedures were delivered by three senior practitioners (each with > 10 years of clinical experience in KOA treatment) who underwent a prestudy calibration session to standardize needle insertion depth, angle, manipulation frequency, and stimulation intensity. Practitioner performance was periodically monitored by the study coordinator to ensure protocol fidelity.

In addition to acupuncture, patients received individualized exercise rehabilitation under the supervision of trained therapists. The program included three components [36, 37]: (1) Knee Joint Extension Training: Patients performed active knee extension exercises, including (a) flexing both knees and gradually extending the affected knee upward, and (b) flexing the affected knee to 90° while keeping the contralateral leg extended, then extending the affected knee with assistance. (2) Muscle Strengthening: Patients engaged in isometric quadriceps contractions by pressing the knee downward against the bed from a flexed position, followed by straight‐leg raises. Training intensity was adjusted based on recovery and tolerance. Each session lasted ~20 min and was performed once daily. (3) Functional Training: Knee Extension: While seated, patients lifted the extended affected leg for 15–20 s, 20 repetitions per session, twice daily. Knee Flexion: In a prone position, patients flexed the knee toward the buttocks while applying ankle traction and simultaneous quadriceps resistance. Each repetition was held for 10 s, 20 repetitions per session, twice daily. All interventions were maintained for 8 weeks, with adherence quantitatively monitored using standardized treatment attendance logs for acupuncture sessions and therapist‐completed checklists for exercise performance. Patients were required to achieve at least 85% session attendance to be considered compliant. Self‐reported home exercise diaries were cross‐validated with weekly therapist interviews to minimize recall bias and ensure data reliability. Of the 151 enrolled participants, all completed the 8‐week intervention protocol, yielding an attrition rate of 0%. Overall compliance, defined as meeting the ≥ 85% attendance threshold, was achieved by 144 patients (95.4%).

### 2.4 Clinical Data Collection

Baseline data were systematically collected prior to the intervention, including demographic variables (body mass index, gender, age), disease‐related characteristics (duration of symptoms, radiographic grade, laterality), prior treatment modalities (medication, physical therapy, acupuncture, injection), clinical assessments (NRS, WOMAC, SF‐12), and hematological parameters (platelets, neutrophils, lymphocytes). Hematological data were obtained from fasting venous blood samples collected between 07:00 and 09:00 on the day of hospital admission before any intervention. Analyses were conducted in the hospital's central laboratory using standardized automated analyzers (Sysmex XN‐1000, Sysmex Corp., Kobe, Japan) with daily internal and external quality control; inter‐ and intra‐assay coefficients of variation were < 5%, ensuring reliable and comparable results. Cases with missing baseline variables were excluded from the final analysis, and no data imputation was performed to avoid introducing bias.

### 2.5 Calculation of SII

The SII was derived from standard hematological indicators routinely measured from peripheral blood at the time of hospital entry. Blood samples were subjected to complete blood count assessment through an automated analyzer, in accordance with established laboratory guidelines. The SII was computed using the formula: neutrophil count multiplied by platelet count divided by lymphocyte count (SII = Neutrophils × Platelet/Lymphocytes), based on absolute values expressed in cells per microliter.

### 2.6 Statistical Analysis

SPSS 22.0 (IBM Corp., Armonk, NY, USA) was used for statistical analysis. A two‐tailed *p* < 0.05 was considered statistically significant. Normality of continuous variables was assessed via the Kolmogorov–Smirnov test. Data were expressed as mean ± standard deviation for normally distributed variables, or median (interquartile range) otherwise. Categorical variables were presented as frequencies and percentages. Group comparisons were performed using the independent samples *t*‐test for continuous variables, which were confirmed to be normally distributed, and the *χ* ^2^ test or Fisher's exact test for categorical variables. Univariate logistic regression was applied to identify baseline clinical and laboratory predictors of treatment efficacy. BMI and lymphocyte count were included as comparison markers because they showed statistically significant differences between responder and nonresponder groups in the independent sample *t*‐test, and both remained statistically significant in univariate logistic regression analysis. Variables with *p* < 0.05 were entered into a multivariate logistic regression model using forward stepwise selection. Results were reported as *β* coefficients, standard errors (SE), Wald *χ* ^2^, *p* values, odds ratios (OR), and 95% confidence intervals (CI). Receiver operating characteristic (ROC) curves were used to assess the predictive performance of SII, BMI, and lymphocyte count. AUC, 95% CI, sensitivity, specificity, and optimal cut‐off values (based on the Youden index) were reported. A combined model incorporating all three variables was also evaluated and compared against individual predictors. All analyses followed established guidelines for clinical prediction modeling and were designed to assess the predictive utility of SII in treatment response among KOA patients undergoing acupuncture and exercise rehabilitation.

## 3 Results

### 3.1 Baseline Characteristics in Effective and Ineffective Groups

The baseline characteristics of patients in the effective and ineffective groups are summarized in Table 1. Of the 151 patients included in this study, 73 were classified into the effective group and 78 into the ineffective group based on clinical response to acupuncture combined with exercise rehabilitation. There was no significant difference in age between the two groups (62.36 ± 5.23 years vs. 63.18 ± 5.08 years, *p* = 0.328). Gender distribution was also comparable (*p* = 0.622), with females accounting for the majority in both the effective (75.34%) and ineffective (71.79%) groups. Disease course duration was similar between groups (6.96 ± 1.55 years vs. 7.21 ± 1.52 years, *p* = 0.329), as was the distribution of radiological grade (*p* = 0.475), with Grade III representing approximately half of each group. Laterality of the affected knee (unilateral vs. bilateral involvement) showed no statistically significant difference (*p* = 0.910), and prior treatment modalities—including medication, physical therapy, acupuncture, and injection—were also not significantly different across groups (*p* = 0.620). However, BMI was significantly lower in the effective group compared to the ineffective group (27.89 ± 1.88 kg/m^2^ vs. 29.79 ± 1.89 kg/m^2^, *p* < 0.001). No statistically significant differences were observed in baseline pain severity, as assessed by the NRS (6.64 ± 0.89 points vs. 6.40 ± 0.93 points, *p* = 0.099), nor in functional status assessed by the WOMAC subscales, including pain (6.60 ± 1.09 points vs. 6.77 ± 0.85 points, *p* = 0.296), stiffness (2.07 ± 0.65 points vs. 2.18 ± 0.70 points, *p* = 0.315), and physical function (20.93 ± 3.11 points vs. 21.40 ± 3.07 points, *p* = 0.355). Similarly, SF‐12 physical and mental component scores did not differ significantly between groups (physical: 30.59 ± 3.96 points vs. 31.28 ± 4.12 points, *p* = 0.294; mental: 50.78 ± 5.23 points vs. 51.42 ± 4.44 points, *p* = 0.417). Analysis of laboratory indices revealed significant differences in systemic inflammatory markers. Although platelet and neutrophil counts were comparable between groups (platelets: 240.36 ± 6.84 vs. 240.83 ± 8.04 × 10^9^/L, *p* = 0.701; neutrophils: 4.07 ± 0.22 vs. 4.13 ± 0.17 × 10^9^/L, *p* = 0.065), lymphocyte count was significantly higher in the effective group (1.97 ± 0.19 vs. 1.83 ± 0.18 × 10^9^/L, *p* < 0.001). Notably, the SII was also significantly lower in the effective group compared to the ineffective group (505.64 ± 10.92 vs. 514.74 ± 9.13 × 10^9^/L, *p* < 0.001), suggesting a potential role of SII as a predictive marker for treatment efficacy.

**Table 1:** Comparison of baseline demographic and clinical characteristics between the ineffective and effective groups in patients with knee osteoarthritis.

Indices	Ineffective group (*n* = 78)	Effective group (*n* = 73)	*p*
Age (years)	63.18 ± 5.08	62.36 ± 5.23	0.328
Gender [*n*(%)]			0.622
Male	22 (28.21)	18 (24.66)	
Female	56 (71.79)	55 (75.34)	
BMI (kg/m^2^)	29.79 ± 1.89	27.89 ± 1.88	< 0.001
Course (years)	7.21 ± 1.52	6.96 ± 1.55	0.329
Radiological grade [*n*(%)]			0.475
Grade II	43 (55.13)	36 (49.32)	
Grade III	35 (44.87)	37 (50.68)	
Affected knee [*n*(%)]			0.91
Unilateral	9 (11.54)	8 (10.96)	
Bilateral	69 (88.46)	65 (89.04)	
Treatment in the past [*n*(%)]			0.62
Medication	43 (55.13)	34 (46.58)	
Physical therapy	20 (25.64)	25 (34.25)	
Acupuncture	13 (16.67)	13 (17.80)	
Injection	2 (2.56)	1 (1.37)	
NRS score	6.40 ± 0.93	6.64 ± 0.89	0.099
WOMAC			
Function subscale	21.40 ± 3.07	20.93 ± 3.11	0.355
Pain subscale	6.77 ± 0.85	6.60 ± 1.09	0.296
Stiffness subscale	2.18 ± 0.70	2.07 ± 0.65	0.315
SF‐12			
Physical health	31.28 ± 4.12	30.59 ± 3.96	0.294
Mental health	51.42 ± 4.44	50.78 ± 5.23	0.417
Laboratory indices			
Platelet (×10^9^/L)	240.83 ± 8.04	240.36 ± 6.84	0.701
Neutrophils (×10^9^/L)	4.13 ± 0.17	4.07 ± 0.22	0.065
Lymphocytes (×10^9^/L)	1.83 ± 0.18	1.97 ± 0.19	< 0.001
SII (×10^9^/L)	514.74 ± 9.13	505.64 ± 10.92	< 0.001

### 3.2 Uni‐ and Multivariate Logistic Regression Analysis Identifying Independent Factors for Predicting the Clinical Efficacy of Acupuncture and Exercise Rehabilitation for Knee Osteoarthritis

**Figure 2:**
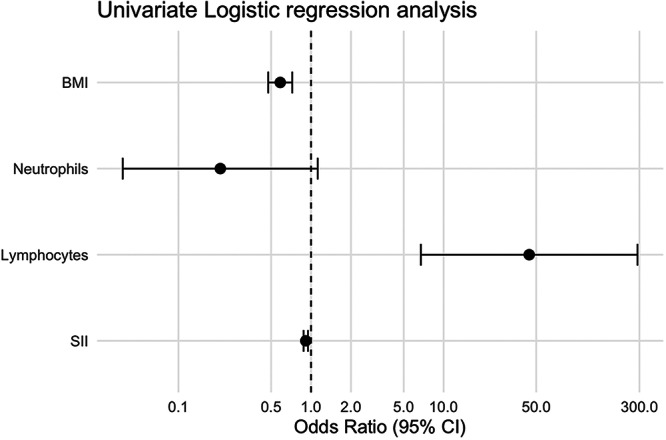
Forest plot of ORs and 95% CIs from univariate logistic regression for predictors of acupuncture plus exercise efficacy in knee osteoarthritis.

To investigate the factors influencing the clinical efficacy of acupuncture combined with exercise rehabilitation in patients with KOA, both univariate and multivariate logistic regression analyses were performed. The univariate logistic regression analysis identified several clinical and inflammatory indices significantly associated with treatment response. As shown in Table 2 and Figure 2, BMI, lymphocyte count, and SII emerged as statistically significant predictors. Specifically, BMI was inversely associated with treatment efficacy (*β* = −0.534, SE = 0.106, Wald *χ* ^2^ = 25.324, *p* < 0.001), with an OR of 0.586 (95% CI: 0.476–0.722), indicating that higher BMI reduced the odds of achieving a favorable clinical response. In contrast, lymphocyte count showed a moderate positive association (*β* = 3.789, SE = 0.960, Wald *χ* ^2^ = 15.729, *p* < 0.001), with an OR of 44.226 (95% CI: 6.736–290.382), suggesting that increased lymphocyte levels may be associated with treatment success. SII was also found to be negatively associated with clinical improvement (*β* = −0.091, SE = 0.020, Wald *χ* ^2^ = 21.783, *p* < 0.001), with an OR of 0.913 (95% CI: 0.878–0.948), implying that higher systemic inflammatory burden may hinder rehabilitation outcomes. Although neutrophil count demonstrated a trend toward negative association (*β* = −1.577, SE = 0.863, Wald *χ* ^2^ = 3.337, *p* = 0.068), this result did not reach statistical significance.

**Table 2:** The predicting factors on the clinical efficacy of acupuncture and exercise rehabilitation for knee osteoarthritis determined by univariate regression analysis.

Clinical indices	*β*	SE	Wald *χ* ^2^	*p*	OR	95% CI
BMI	−0.534	0.106	25.324	< 0.001	0.586	0.476~0.722
Neutrophils	−1.577	0.863	3.337	0.068	0.207	0.038~1.122
Lymphocytes	3.789	0.96	15.729	< 0.001	44.226	6.736~290.382
SII	−0.091	0.02	21.783	< 0.001	0.913	0.878~0.948

**Figure 3:**
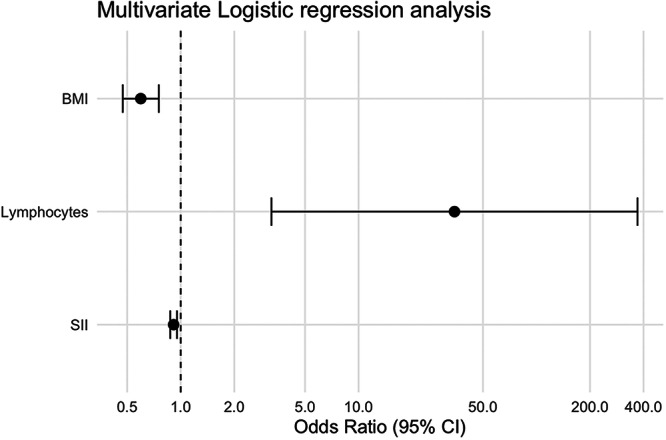
Forest plot of ORs and 95% CIs from multivariate logistic regression for predictors of acupuncture plus exercise efficacy in knee osteoarthritis.

To further determine the independent predictors of treatment efficacy, variables found to be significant in the univariate analysis were included in a multivariate logistic regression model. As shown in Table 3 and Figure 3, BMI, lymphocyte count, and SII remained statistically significant independent predictors of clinical outcome. In the multivariate model, BMI continued to exhibit a significant negative association (*β* = −0.518, SE = 0.119, Wald *χ* ^2^ = 18.793, *p* < 0.001), with an adjusted OR of 0.596 (95% CI: 0.472–0.753). Lymphocyte count remained a moderate independent positive predictor of clinical efficacy (*β* = 3.544, SE = 1.209, Wald *χ* ^2^ = 8.588, *p* = 0.003), with an adjusted OR of 34.597 (95% CI: 3.234–370.131). Notably, SII continued to show an independent inverse association with treatment success (*β* = −0.092, SE = 0.023, Wald *χ* ^2^ = 16.576, *p* < 0.001), with an adjusted OR of 0.912 (95% CI: 0.873–0.953), reinforcing the potential value of SII as a predictive biomarker in the management of KOA.

**Table 3:** The predicting factors on the clinical efficacy of acupuncture and exercise rehabilitation for knee osteoarthritis determined by multivariate regression analysis.

Clinical indices	*β*	SE	Wald *χ* ^2^	*p*	OR	95% CI
BMI	−0.518	0.119	18.793	< 0.001	0.596	0.472~0.753
Lymphocytes	3.544	1.209	8.588	0.003	34.597	3.234~370.131
SII	−0.092	0.023	16.576	< 0.001	0.912	0.873~0.953

### 3.3 The ROC Analysis Evaluating the Predicting Effect of SII on the Clinical Efficacy of Acupuncture and Exercise Rehabilitation for Knee Osteoarthritis

**Figure 4:**
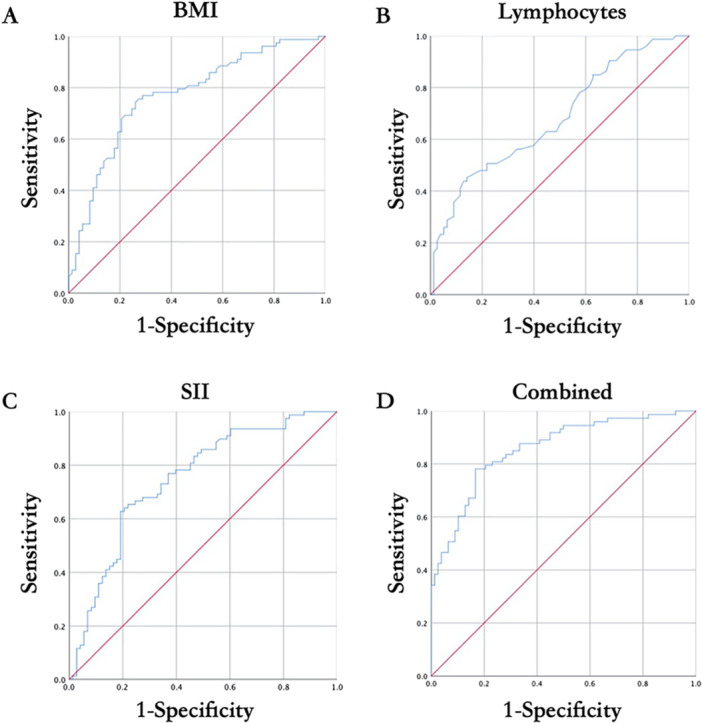
ROC curve evaluating the predicting effects of (A) BMI, (B) lymphocytes, (C) SII, and (D) combined BMI + lymphocytes + SII analysis on the clinical efficacy of acupuncture and exercise rehabilitation for knee osteoarthritis.

To further evaluate the clinical utility of the SII in predicting the therapeutic response to acupuncture combined with exercise rehabilitation in patients with KOA, ROC curve analysis was conducted (Table 4 and Figure 4). As summarized in Table 4, SII exhibited an acceptable discriminative performance, with an AUC of 0.749 (95% CI: 0.670–0.828, *p* < 0.001). The optimal cut‐off value for SII was determined to be 512.14, which corresponded to a sensitivity of 64.1% and a specificity of 79.5% for predicting clinical efficacy. In addition to SII, BMI also demonstrated a robust predictive value, with an AUC of 0.770 (95% CI: 0.694–0.845, *p* < 0.001). The best threshold value of 28.755 kg/m^2^ yielded a sensitivity of 74.4% and a specificity of 74.0%, indicating a balanced performance in identifying responders and nonresponders. Lymphocyte count showed a relatively modest predictive capability, with an AUC of 0.683 (95% CI: 0.598–0.767, *p* < 0.001). At the optimal cut‐off value of 1.985 × 10^9^/L, the sensitivity was 45.2%, whereas the specificity reached 85.9%, suggesting that while lymphocyte count may be more useful in confirming likely responders, its standalone sensitivity is limited. Pairwise AUC comparisons further revealed that BMI performed significantly better than lymphocyte count (*p* < 0.001), while no statistically significant difference was observed between BMI and SII (*p* = 0.711). Notably, SII outperformed lymphocyte count with a statistically significant margin (*p* < 0.001), underscoring its relative advantage over conventional hematological parameters in this setting. Importantly, when SII, BMI, and lymphocyte count were combined into a composite predictive model, the overall diagnostic performance improved markedly. The combined model yielded an AUC of 0.858 (95% CI: 0.799–0.917, *p* < 0.001), with a specificity of 83.3% and a sensitivity of 78.1%. These results underscore the value of SII as an accessible and effective biomarker, particularly when used in conjunction with BMI and lymphocyte count, to predict clinical benefit from acupuncture and exercise rehabilitation in KOA patients.

**Table 4:** The ROC analysis evaluating the effects of SII on the prediction of clinical efficacy of acupuncture and exercise rehabilitation for knee osteoarthritis.

Variables	AUC	95% CI	Best cut‐off value	Sensitivity (%)	Specificity (%)	*p*	*p* values for pairwise AUC comparisons
BMI	0.77	0.694~0.845	28.755	74.4	74.0	< 0.001	BMI vs. lymphocytes: *p* < 0.001
							BMI vs. SII: *p* = 0.711
Lymphocytes	0.683	0.598~0.767	1.985	45.2	85.9	< 0.001	Lymphocytes vs. SII: *p* < 0.001
SII	0.749	0.670~0.828	512.14	64.1	79.5	< 0.001	
Combined	0.858	0.799~0.917		78.1	83.3	< 0.001	

## 4 Discussion

In this retrospective study of 151 patients with KOA undergoing acupuncture combined with exercise rehabilitation, we identified SII, BMI, and lymphocyte count as independent predictors of clinical efficacy. Both univariate and multivariate logistic regression analyses demonstrated that lower SII and BMI, as well as higher lymphocyte counts, were significantly associated with favorable treatment outcomes. ROC curve analysis further confirmed the predictive utility of these indices, with SII (AUC = 0.749) performing comparably to BMI (AUC = 0.770) and outperforming lymphocyte count (AUC = 0.683). Importantly, a composite model incorporating SII, BMI, and lymphocyte count achieved the highest discriminative ability (AUC = 0.858), highlighting the potential value of combining clinical and inflammatory markers to guide individualized treatment planning for KOA.

Our data revealed a significantly lower SII in patients who responded favorably to the combined therapy, underscoring the importance of systemic inflammatory status in influencing treatment outcomes. SII is a composite marker derived from neutrophil, platelet, and lymphocyte counts, and reflects the balance between the pro‐inflammatory and immune‐regulatory arms of the immune system [38, 39]. Elevated neutrophils and platelets are typically associated with heightened inflammatory activity, which contributes to synovial hyperplasia, cartilage breakdown, and pain sensitization in KOA [40, 41]. In contrast, lymphocytes, particularly regulatory T cells and Th2 cells, play key roles in dampening inflammation, facilitating tissue repair, and maintaining immune homeostasis [42, 43]. A higher SII suggests a predominance of pro‐inflammatory processes and reduced immunomodulatory capacity, which may impair the physiological mechanisms through which acupuncture and exercise exert their therapeutic effects. Although this study did not perform a formal cost‐effectiveness analysis, the predictive factors identified, particularly SII, are derived from routine blood tests that are inexpensive, widely available, and easily integrated into standard clinical workflows. This enhances the clinical practicality of applying our findings, especially in resource‐limited settings, and supports their potential for broader implementation in real‐world KOA management.

The biological mechanisms linking inflammation to reduced responsiveness to acupuncture and exercise are likely multifaceted. Acupuncture has been shown to modulate central and peripheral pathways involved in pain perception and inflammatory regulation [10, 44, 45]. Mechanistically, it activates the hypothalamic–pituitary–adrenal axis and enhances the release of anti‐inflammatory cytokines (e.g., IL‐10), endogenous opioids, and neuropeptides such as substance P and CGRP [46, 47]. These processes may be blunted in the presence of elevated systemic inflammation, which can disrupt neuroimmune signaling and reduce sensitivity to external modulatory inputs [48]. Likewise, chronic inflammation impairs neuromuscular function, reduces muscle protein synthesis, and alters proprioceptive feedback, factors critical for the success of exercise rehabilitation [49]. Therefore, a lower SII may serve as a surrogate for a more favorable immunological milieu, conducive to the full engagement of these therapeutic mechanisms.

In addition to its role as a therapeutic technique, acupuncture is deeply rooted in the theoretical framework of TCM, particularly in the field of Traditional Chinese Orthopedics [50, 51]. Within this framework, KOA is generally categorized under the syndrome of “Bi” (impediment), which is primarily attributed to obstruction of meridians and collaterals by wind, cold, dampness, or heat, leading to stagnation of qi and blood and subsequent pain, stiffness, and limited mobility [52, 53]. TCM diagnostic thinking emphasizes individualized treatment based on syndrome differentiation, such as cold‐damp obstruction, damp‐heat accumulation, or liver‐kidney deficiency, with the therapeutic principle of “dispelling pathogenic factors and unblocking channels” [54]. Acupuncture point selection in this study, such as Liangqiu (ST34), Xuehai (SP10), and Yanglingquan (GB34), is consistent with standard TCM practice for invigorating blood circulation, resolving dampness, and relaxing tendons to restore joint function [54]. Integrating these TCM concepts into modern clinical research not only supports the rationale for acupuncture point prescription but also bridges traditional pathogenesis theory with measurable biomedical outcomes such as SII, potentially providing a more comprehensive framework for understanding and optimizing KOA management.

Another important finding was the strong inverse relationship between BMI and treatment efficacy. High BMI is not only a mechanical load factor but also a major driver of systemic low‐grade inflammation, mediated through adipokines such as leptin, resistin, and pro‐inflammatory interleukins [41, 55, 56]. These adipose‐derived mediators amplify joint inflammation, induce chondrocyte catabolism, and impair cartilage regeneration [57, 58]. Furthermore, obesity is associated with impaired muscle function, reduced exercise tolerance, and joint malalignment, all of which may compromise the efficacy of physical rehabilitation [59, 60]. Notably, BMI alone may not fully capture variations in body composition that influence functional recovery. For example, sarcopenic obesity, characterized by increased fat mass with concurrent loss of skeletal muscle, has been linked to poorer mobility, reduced muscle strength, and greater disability in KOA patients, even at similar BMI levels [61, 62]. According to the study implemented by Godziuk et al. [62], sarcopenic obesity in adults with end‐stage KOA is consistently associated with reduced walking speed, endurance, muscle strength, and greater difficulty in self‐care activities, highlighting the need for combined diagnostic approaches for early identification and management. This phenotype may further attenuate the benefits of exercise rehabilitation and acupuncture by limiting functional reserve and adaptability. It is also plausible that increased adiposity alters the pharmacodynamics of acupuncture by modifying subcutaneous fat thickness and tissue perfusion, thereby diminishing its physiological effects. These findings highlight the need for integrative management strategies that include weight control and metabolic regulation in overweight or obese KOA patients undergoing nonpharmacological therapies.

Lymphocyte count, which positively correlated with treatment response, further supports the role of immune competence in modulating rehabilitation outcomes. Lymphocytes are central to adaptive immunity and tissue homeostasis [42, 63, 64]. Their role in regulating pro‐inflammatory cytokine release, promoting angiogenesis, and facilitating resolution of inflammation suggests that higher lymphocyte levels may reflect a more competent and regulated immune environment, which can better synergize with the anti‐inflammatory and regenerative effects of acupuncture and exercise. In addition, evidence suggests that lymphocyte‐mediated modulation of synovial inflammation may influence the sensitivity of nociceptors and, by extension, pain perception, one of the core targets of acupuncture therapy [65, 66, 67]. However, in our analysis, although lymphocyte count emerged as a statistically significant predictor of treatment response, its sensitivity was relatively low (45.2%), indicating that many responders would not be correctly identified if lymphocyte count were used in isolation. This limitation suggests that lymphocyte count may have restricted standalone predictive value and should be interpreted in conjunction with other markers such as SII and BMI to improve overall diagnostic performance. The inclusion of lymphocyte count in a combined predictive model may help to offset its low sensitivity while leveraging its specificity to refine patient stratification.

The ROC analysis supported the utility of these biomarkers in predicting treatment efficacy. SII demonstrated a good area under the curve (AUC = 0.749), indicating its practical value as a discriminative tool. Although BMI yielded a slightly higher AUC (0.770), it is notable that SII reflects an underlying inflammatory state that may be modifiable through intervention, whereas BMI typically requires longer‐term lifestyle modification. Lymphocyte count alone had a lower AUC (0.683), but with high specificity, suggesting its value in confirming rather than screening for likely responders. Most importantly, the composite model integrating SII, BMI, and lymphocyte count achieved the highest AUC (0.858), indicating that the combined assessment of immunological and metabolic parameters provides superior predictive accuracy compared to any single variable. This supports a multibiomarker approach to patient stratification in KOA rehabilitation programs. The combining predictive roles of SII, BMI, and lymphocyte count may be biologically interconnected. Higher BMI is often accompanied by increased visceral adiposity, which promotes chronic low‐grade inflammation via adipokine secretion and pro‐inflammatory cytokine release [68, 69]. This inflammatory milieu can elevate neutrophil counts, suppress lymphocyte‐mediated immune regulation, and consequently raise SII levels. Conversely, higher lymphocyte counts may reflect a more balanced immune response capable of counteracting obesity‐related inflammation, thereby improving the likelihood of a favorable therapeutic outcome [70]. These interrelationships suggest that SII may partially mediate the impact of BMI and lymphocyte status on treatment efficacy, highlighting the value of integrating these parameters for more precise patient stratification.

The absence of significant differences in traditional clinical and demographic parameters, such as age, sex, symptom duration, Kellgren–Lawrence grade, and baseline NRS/WOMAC scores, between responders and nonresponders emphasizes the limitations of relying solely on clinical presentation or radiographic severity when predicting treatment outcomes. It suggests that systemic biological factors may exert a more profound influence on treatment responsiveness than the degree of joint structural damage or self‐reported symptoms at baseline. This reinforces the need for objective biomarkers, such as SII, to enhance clinical decision‐making and facilitate precision medicine in KOA.

This study has several limitations. Its retrospective design, though useful for hypothesis generation, is prone to confounding and selection bias, and residual effects from unmeasured variables cannot be excluded despite multivariate adjustment. The retrospective single‐center design limits the ability to establish causal relationships and increases the risk of selection bias. These issues will be addressed in future prospective multicenter studies, which will strengthen causal inferences and reduce selection bias. Being single‐center, generalizability is limited. Longitudinal tracking of SII and other immune markers, along with prospective studies incorporating cytokine profiles, T‐cell subsets, and immunophenotyping, could clarify temporal relationships with symptom change. Stratified analysis by KL Grade II versus III was not done due to small sample size but will be addressed in larger cohorts. The proposed combined prognostic model has not undergone external validation in an independent sample, which reduces its potential clinical applicability. Future research will focus on validating this model in independent, larger cohorts to enhance its clinical relevance and generalizability. Findings should be cautiously generalized beyond this Chinese tertiary hospital, as differences in treatment protocols, practitioner expertise, patient expectations, healthcare access, reimbursement, and integration of TCM may influence adherence and outcomes. Future multicenter, cross‐national research is needed to validate SII's predictive value. Finally, using MCII as a dichotomous outcome may oversimplify improvement and misclassify borderline cases; however, this approach was chosen to provide a clear and clinically interpretable definition of treatment success based on established thresholds of meaningful improvement, and continuous or ordinal measures may better capture the full response spectrum.

## 5 Conclusion

In conclusion, this study suggests that SII represents a relatively practical and readily obtainable marker for estimating clinical efficacy in KOA patients undergoing acupuncture and exercise rehabilitation. Together with BMI and lymphocyte count, SII offers a promising biomarker for stratifying patients, optimizing therapeutic strategies, and improving clinical outcomes. These findings underscore the importance of systemic inflammatory and metabolic status in determining treatment responsiveness and provide a foundation for integrating biological markers into individualized care pathways for KOA. Further prospective validation and mechanistic exploration are warranted to consolidate the clinical utility of SII in this context. However, given the retrospective single‐center design, modest AUC values, and lack of external validation, these results should be interpreted as preliminary. Future multicenter, prospective studies with larger and more diverse cohorts, as well as mechanistic investigations, are warranted to further confirm and refine the clinical utility of SII in this context.

## Author Contributions

Conceptualization: Bin Shi. Supervision: Bin Shi. Funding acquisition: Bin Shi. Validation: Shifei Sun, Zhiyong Lu, and Qing Yang. Investigation: Wenchang Xu and Fengjun Zhang. Formal analysis: Shifei Sun and Gongchang Yu. Methodology: Zhiyong Lu and Qing Yang. Writing – original draft: Shifei Sun. Writing – review and editing: Bin Shi, Zhiyong Lu, and Qing Yang. All the authors read and approved the final manuscript.

## Ethics Statement

This study was conducted in accordance with the 1964 Declaration of Helsinki and its later amendments or comparable ethical standards. Ethical approval was obtained by the Ethics Committee of Neck‐Shoulder and Lumbocrural Pain Hospital of Shandong First Medical University.

## Consent

All participants signed written informed consent.

## Conflicts of Interest

The authors declare no conflicts of interest.

## Supporting information

Supporting File

## Data Availability

The data set generated and analyzed during the current study is available from the corresponding author upon reasonable request.
